# Supplementary material for: Human intestinal nematode infections in Sri Lanka: A scoping review
Source: PLoS Negl Trop Dis. 2024 Dec 2;18(12):e0012689. doi: 10.1371/journal.pntd.0012689 (PMC11637441; doi:10.1371/journal.pntd.0012689)
Supplement: S1 Table — (PDF) [file pntd.0012689.s002.pdf]

S1 Table. Quality assessment of epidemiological studies from the year 2000, based on the modified Newcastle-Ottawa Quality Assessment Scale (1)

		Selection				Comparability	Outcome				
**Study No**	**First Author, Year of publication**	Representativeness of the sample	Ascertainment of specimen collection methods	Sample size	Non-respondents	Impact of Bias (selection bias, measurement bias, participant reporting, confounders)	Assessment of the outcome (STH infection)	Statistical analysis	**Total Score (Out of 10)**	**Quality Assurance Grade**	**References**
**1**	Fernando et al, 2000	2	1	1	1	0	2	1	8	Good	(2)
**2**	Fernando et al, 2001	2	1	1	1	0	2	1	8	Good	(3)
**3**	Selvaratnam et al, 2003	2	1	1	1	0	2	1	8	Good	(4)
**4**	De Silva et al, 2003	2	1	1	1	1	2	1	9	Very good	(5)
**5**	Chandrasena et al, 2004	2	1	1	1	1	2	1	9	Very good	(6)
**6**	Gunawardena et al, 2004	2	1	1	1	0	2	1	8	Good	(7)
**7**	Pathmeswaran et al, 2005	2	1	1	1	1	2	1	9	Very good	(8)
**8**	Banneheka et al, 2006	2	1	I	1	0	2	1	8	Good	(9)
**9**	Chandrasena et al, 2007	2	1	1	1	0	2	1	8	Good	(10)
**10**	Gunawardena et al, 2008	2	1	1	1	1	2	1	9	Very good	(11)
**11**	Chandrasena et al, 2010	2	1	1	1	1	2	1	9	Very good	(12)
**12**	Kumarendran et al, 2010	2	1	1	1	0	2	1	8	Good	(13)
**13**	Gunawardena et al, 2010	2	1	1	1	1	2	1	9	Very good	(14)
**14**	Gunawardena et al, 2011	2	1	1	1	0	2	1	8	Good	(15)
**15**	Rathnayaka et al, 2012	2	1	1	1	0	2	1	8	Good	(16)
**16**	Kurnithas et al, 2012	2	1	1	1	1	2	1	9	Very good	(17)
**17**	Gunawardena et al, 2013	2	1	1	1	0	2	1	8	Good	(18)
**18**	Gunawardena et al, 2013	2	1	1	1	0	2	1	8	Good	(19)
**19**	Gunawardena et al, 2014	2	1	1	1	1	2	1	9	Very good	(20)
**20**	Suraweera et al, 2015	2	1	1	1	1	2	1	9	Very good	(21)
**21**	Galgamuwa et al, 2016	2	1	1	1	0	2	1	8	Good	(22)
**22**	Kumarendran et al, 2017	2	1	1	1	0	2	1	8	Good	(23)
**23**	Galgamuwa et al, 2017	2	1	1	1	1	2	1	9	Very good	(24)

**24**	Lepper et al, 2018	2	1	1	1	1	2	1	9	Very good	(25)
**25**	Ubayawardena et al, 2018	2	1	1	1	0	2	1	8	Good	(26)
**26**	Galgamuwa et al, 2018	2	1	1	1	1	2	1	9	Very good	(27)
**27**	Suraweera et al, 2018	2	1	1	1	0	2	1	8	Good	(28)
**28**	Ediriweera et al, 2019	2	1	1	1	2	2	1	10	Very good	(29)

References

1. Wells GA, Shea B, O'Connell Da, Peterson J, Welch V, Losos M, et al. The Newcastle-Ottawa Scale (NOS) for assessing the quality of nonrandomised studies in meta-analyses. Oxford; 2000.Available from: https://www.researchgate.net/publication/261773681
2. Fernando SD, Paranavitane SR, Rajakaruna J, Weerasinghe S, Silva D, Wickremasinghe AR. The health and nutritional status of school children in two rural communities in Sri Lanka. Trop Med Int Health. 2000;5(6):450-2. doi: 10.1046/j.1365-3156.2000.00569.x.
3. Fernando SD, Goonethilleke H, Weerasena KH, Kuruppuarachchi ND, Tilakaratne D, de Silva D, Wickremasinghe AR. Geo-helminth infections in a rural area of Sri Lanka. Southeast Asian J Trop Med Public Health. 2001;32(1):23-6. PMID: 11485089.
4. Selvaratnam RR, de Silva LD, Pathmeswaran A, de Silva NR. Nutritional status and productivity of Sri Lankan tea pluckers. Ceylon Med J. 2003; 48(4):114-8. doi: 10.4038/cmj.v48i4.3326.
5. De Silva NR, Pathmeswaran A, Fernando SD, Weerasinghe CR, Selvaratnam RR, Padmasiri EA, Montresor A. Impact of mass chemotherapy for the control of filariasis on geohelminth infections in Sri Lanka. Ann Trop Med Parasitol. 2003;97(4):421-5. doi: 10.1179/000349803235002407.

6. Chandrasena TG, de Alwis AC, de Silva LD, Morel RP, de Silva NR. Intestinal parasitoses and the nutritional status of Veddah children in Sri Lanka. Southeast Asian J Trop Med Public Health. 2004;35(2):255-9. PMID: 15691120.

7. Gunawardena GSA, Karunaweera ND, Ismail MM. A study on the seasonal variation in the incidence of *Ascaris* infection in the plantation sector of Sri Lanka. In: Collected papers on the control of soil-transmitted helminthiases. Asian Parasite Control Organization, Eds. Hayashi, S et al., 2001,VII: 104 - 112.

8. Pathmeswaran A, Jayatissa R, Samarasinghe S, Fernando A, de Silva RP, Thattil RO, de Silva NR. Health status of primary schoolchildren in Sri Lanka. Ceylon Med J. 2005;50(2):46-50. doi: 10.4038/cmj.v50i2.1567.

9. Banneheke BMHA, Gunawardane DMDE, Pinidiyapathirage M, Ekanayake S, Withana R, Tilakaratne WD. Geohelminthic infection in an estate child population in Sri Lanka: association with hygienic practices and eosinophil counts. Bull Sri Lanka Coll Microbiol. 2006;04(1):16. Available from: tp://repository.kln.ac.lk/handle/123456789/13305

10. Chandrasena TG, Hapuarachchi HC, Dayanath MY, Pathmeswaran A, de Silva NR. Intestinal parasites and the growth status of internally displaced children in Sri Lanka. Trop Doct. 2007;37(3):163-5. doi: 10.1258/004947507781524610.

11. Gunawardena NK, Amarasekera ND, Pathmeswaran A, de Silva NR. Effect of repeated mass chemotherapy for filariasis control on soil-transmitted helminth infections in Sri Lanka. Ceylon Med J. 2008 Mar;53(1):13-6. doi: 10.4038/cmj.v53i1.220.

12. Chandrasena TG, Balasooriya BA, Imbulpitiya IV, de Silva NR. A survey for intestinal parasites in a psychiatric institution in Sri Lanka. Ann Trop Med Parasitol. 2010;104(7):605-8. doi: 10.1179/136485910X12786389891605.

13. Kumarendran B. Soil-transmitted helminth infections among plantation sector primary school children in Nuwara Eliya District. Available

from: http://192.248.21.144/handle/1/1343

14. Gunawardena NK, Gunasingha HMMSK, Kumarendran B, Pathmeswaran A, de Silva NR. Prevalence of soil-transmitted helminth infections and malnutrition among 9-10 year old children attending estate sector schools. The Ceylon Medical Journal. 2010; 55(Supplement 1):28. Available from: http://repository.kln.ac.lk/handle/123456789/9889

15. Gunawardena K, Kumarendran B, Ebenezer R, Gunasingha MS, Pathmeswaran A, de Silva N. Soil-transmitted helminth infections among plantation sector schoolchildren in Sri Lanka: prevalence after ten years of preventive chemotherapy. PLoS Negl Trop Dis. 2011;5(9):e1341. doi: 10.1371/journal.pntd.0001341.

16. Rathnayaka RMK, Wang. Prevalence and effect of personal hygiene on the transmission of helminth infection among primary school children living in slums. Int J Multidiscip Res Rev. 2012;2(7):1–13. Available from: https://www.researchgate.net/publication/317064458_prevalence_and_effect_of_personal_hygiene_ontransmission_of_helminthes_infection_among_primary_school_children_living_in_slums

17. Rasaratnam K, Murugananthan A, Kannathasan S. Prevalence and associated factors of soil transmitted helminthes infestation among preschool children of Vadamaradchi Educational Zone. Ving. Journal of Science 2011;10(1):25-34. doi: 10.4038/vingnanam.v10i1.4075

18. Gunawardena NK, Chandrasena TN, de Silva NR. Prevalence of enterobiasis among primary school children in Ragama, Sri Lanka. Ceylon Med J. 2013;58(3):106-10. doi: 10.4038/cmj.v58i3.5039.

19. Gunawardena S, Gunatilleke H, Ismail M. Prevalence of *Enterobius vermicularis* infection among schoolchildren attending four selected

schools in the Hambantota district of Sri Lanka. Sri Lankan J Infect Dis. 2013;3(2):17-20. doi: 10.4038/sljid.v3i2.5467

20. Suraweera O, Galgamuwa L, Iddawela D, Wickramasinghe S. Prevalence and associated factors of *Enterobius vermicularis* infection in children from a poor urban community in Sri Lanka: a cross-sectional study. Int J Res Med Sci. 2015; 3(8):1994-1999. doi: 10.18203/2320-6012.ijrms20150315

21. Gunawardena S, Gunawardena NK, Kahathuduwa G, Karunaweera ND, de Silva NR, Ranasinghe UB, Samarasekara SD, Nagodavithana KC, Rao RU, Rebollo MP, Weil GJ. Integrated school-based surveillance for soil-transmitted helminth infections and lymphatic filariasis in Gampaha district, Sri Lanka. Am J Trop Med Hyg. 2014;90(4):661-6. doi: 10.4269/ajtmh.13-0641.

22. Galgamuwa L, Iddawela D, Dharmaratne SD. Factors associated with the prevalence of *Ascaris lumbricoides* infection among preschool children in a plantation community, Kandy district, Sri Lanka. Southeast Asian J Trop Med Public Health. 2016 Nov;47(6):1143-52. PMID: 29634161.

23. Kumarendran B, Pathmeswaran A, NR D. Prevalence of pinworm infection among children living in low income settlements in Colombo municipal council area. Sri Lanka Medical Association, 130th Anniversary International Medical Congress.2017;62(Supplement 1):94

24. Galgamuwa LS, Iddawela D, Dharmaratne SD. Prevalence and intensity of *Ascaris lumbricoides* infections in relation to undernutrition among children in a tea plantation community, Sri Lanka: a cross-sectional study. BMC Pediatr. 2018:25;18(1):13. doi: 10.1186/s12887-018-0984-3.

25. Lepper HC, Prada JM, Davis EL, Gunawardena SA, Hollingsworth TD. Complex interactions in soil-transmitted helminth co-infections from a cross-sectional study in Sri Lanka. Trans R Soc Trop Med Hyg. 2018;112(8):397-404. doi: 10.1093/trstmh/try068.

26. Ubhayawardana N, Gammana Liyanage I, Herath HMJCB, Amarasekera U, Dissanayake T, de Silva S, Fernando N, Ekanayake S. Direct

microscopy of stool samples for determining the prevalence of soil-transmitted helminthic infections among primary school children in Kaduwela MOH area of Sri Lanka following floods in 2016. J Environ Public Health. 2018;2018:4929805. doi: 10.1155/2018/4929805.

27. Galgamuwa LS, Iddawela D, Dharmaratne SD. Association between intestinal helminth infections and mid-upper-arm circumference among children in Sri Lanka: a cross-sectional study. J Helminthol. 2018; 92(3):291-7. doi: 10.1017/S0022149X17000517.

28. Suraweera O, Galgamuwa L, Wickramasinghe S, Iddawela D, Nandasiri N. Soil-transmitted helminth infections, associated factors and nutritional status in an estate community in Sri Lanka. Sri Lankan J Infect Dis. 2018;8(2):100. doi: 10.4038/sljid.v8i2.8226

29. Ediriweera DS, Gunawardena S, Gunawardena NK, Iddawela D, Kannathasan S, Murugananthan A, et al. (2019) Reassessment of the prevalence of soil-transmitted helminth infections in Sri Lanka to enable a more focused control programme: a cross-sectional national school survey with spatial modelling. Lancet Glob Heal. 2019;7(9):e1237–46. doi.org/10.1016/S2214-109X(19)30253-0
