# Supplementary material for: Human intestinal nematode infections in Sri Lanka: A scoping review
Source: PLoS Negl Trop Dis. 2024 Dec 2;18(12):e0012689. doi: 10.1371/journal.pntd.0012689 (PMC11637441; doi:10.1371/journal.pntd.0012689)
Supplement: Attachment — Submitted filename: Response to reviewers.docx [file pntd.0012689.s003.docx]

**Reviewers' comments:**

***We sincerely appreciate both the reviewers for their constructive comments and useful insights, which were found to be invaluable in improving our scoping review.***

***Rsponse to reviewer #* 1**

Reviewer #1: This paper could serve as a useful review of HINI trends in Sri Lanka over the last 50 years, encompassing the recent civil war, MDG period and beyond. However, it would need to be much sharper and focused on quality information

1. Reviewer #1: This extensive review has perused a large volume of information on human intestinal nematode infections (HINI) in Sri Lanka. While the methods list the vastly disparate information perused, with data going back to the 19th century, it becomes impossible to ascertain study quality and relevance. I would have proposed restricting the review to the last 3-4 decades or maximally, the post-independence period.

***Response – We have revised the key analysis focusing on the prevalence and trends within the last two decades presenting a deeper context. Quality of all the prevalence assessment studies of the last two decades were done using the modified Newcastle-Ottawa Quality Assessment tool ensuring the quality of the data presented in the study (Provided in the supplementary file Table 2). We have also provided a detailed discussion on the implication of our findings in the context of public health policy, research gaps and future directions for the research. (Lines 282-438).***

2. The tables too, while indicating the diagnostic methods used, do not provide information on sample size, or sampling frame and include facility-based studies. The review should ideally be restricted to population-based surveys excluding retrospective analyses or case series, which though excluded in the flow diagram, still seem to be included in the tables.

***Response – We have revised the prevalence assessment tables to include the sample size, category of the population, the parasite species types, and the diagnostic method/s employed. In discussing the table information, we have clarified that all the studies are conducted as either cross-sectional studies or islandwide surveys. Following the suggestion of the second reviewer, we have incorporated certain publications such as reviews, into the discussion. (Lines 320-387 and Table 2)***

3. Tables and figures are informative, they could be much improved by only focusing on reasonable population representative information and reasonable size studies. Also, a more recent set of studies would better epidemiological and policy sense

***Response – As suggested we have focused the prevalence analysis section of the review to the last two decades. We have given the sample sizes of the studies. The quality of the studies was assessed using the modified Newcastle-Ottawa Quality Assessment tool ensuring the quality of the data presented in the study (Provided in the supplementary file Table 2). ( Lines 320-393)***

***We have also provided a detailed discussion on the implication of our findings in the context of public health policy, research gaps and future directions for the research. (Lines 282-438).***

4. The paper could benefit from a robust discussion on limitations, such as the relationship of any temporal trends associated with HINI prevalence and control programs, periods of socio-economic growth and investments in WASH

***Response – As suggested we have discussed the limitations of the study (Lines 512-523), the relationship of temporal trends with prevalence and control programmes (Lines 388-398) and the period of socioeconomic growth and investment in WASH. (Lines 412-430)***

***Response to reviewer #* 2**

Reviewer #2: The manuscript titled "Human intestinal nematode infections in Sri Lanka: a scoping review" is a comprehensive research article focused on the prevalence, diagnostic methods, and infections of human intestinal nematodes in Sri Lanka. The abstract highlights a significant reduction in infection prevalence due to control programs but notes limited data on the distribution, trends, and challenges of these infections.

The methodology involves a systematic review of published information and grey literature, adhering to PRISMA-ScR standards. The findings indicate a historical presence of these infections in Sri Lanka, with a notable decline in prevalence over time. However, issues like underreporting due to diagnostic methods and persisting public health problems in specific communities are raised.

The conclusion suggests the need for tailored intervention strategies for different regions and highlights the potential of Sri Lanka's experience to inform other countries facing similar challenges.

1. Clarity and Structure: The manuscript is well-structured with clear sections. However, some parts, particularly in the methodology and results, could benefit from more concise language and better organization for easier readability. See Reviewer Attachment.

***Response - As proposed, we have revised the methodology and results to make it more concise and better organized. (Lines 139-219)***

**Comments in the attachment**

1.1 Any other or no population criteria

***Response - Inclusion criteria were revised (Lines 198-209)***

1.2 Authors should state the reason for excluding review papers that may address some of the objectives of this scoping review. Would have contributed to history of disease and geopolitical distribution and other narrative segments of this scoping review.

***Response - We have revised the exclusion criteria to incorporate reviews, reports, and letters to editors containing relevance to the objectives of the scoping review. (Lines 210-215)***

1.3 Who were the reviewers?

***Response - We have stated the initials of the reviewers who have done the study selection and data extraction (Lines 188-197)***

2. Methodological Rigor: The methodology follows PRISMA-ScR standards, which is commendable. However, the review could be strengthened by providing more details on the reasons for the inclusion and exclusion criteria, how disagreements between reviewers were managed and a more detailed description of the data extraction process.

***Response - As suggested we have refined the inclusion and exclusion criteria to provide comprehensive details on both inclusions and exclusions. Additionally, we have described how the disagreements between reviewers were managed. (Lines188-215)***

3. Comprehensiveness of Literature Review: The review appears extensive, but it's unclear if it encompasses all relevant literature, including non-English sources.

***Response – Even though we have not put any language restrictions we did not find any native language articles on human intestinal nematode infections in Sri Lanka. We have stated it in the manuscript. (Lines 241-242)***

4. Analysis of Findings: While the review presents the data collected, it lacks depth in the analysis. A more detailed discussion on the implications of the findings, especially in the context of public health policy and future research directions, would be valuable.

***Response – We have revised the key analysis focusing on the prevalence and trend analysis over the last two decades presenting a deeper context. We have also provided a detailed discussion on the implication of our findings in the context of public health policy, research gaps and future directions for the research. (Lines 320-393)***

5. Discussion of Socio-economic Factors: The manuscript could benefit from a more detailed discussion on the socio-economic determinants of infection prevalence and how they affect control strategies.

***Response – We have discussed the socioeconomic factors determining infection prevalence and and their impact on control strategies. This includes an analysis of how periods of economic growth in the country have facilitated infection control measures, as well as a discussion on the repercussions of the recent economic crisis on prevalence of human intestinal nematode infections. (Lines 393-444)***

6. Regional Comparisons and Global Context: The manuscript could be enriched by comparing Sri Lanka's situation with similar contexts in other countries in Asia and the rest of the world, to provide a more regional or global perspective on the issue.

***Response – As proposed, we have compared the Sri Lankan context of human intestinal nematode infections with other countries, with a particular focus on the Asian region. (Lines 445-478)***

7. Recommendations for Future Research: The Discussion section could offer more specific directions for future research, such as identifying gaps in the current understanding of the prevalence and control of these infections.

***Our response – As suggested, we have discussed the research gaps identified during the review and the directions for future research. (Lines 457-505)***

8. Practical Implications and Policy Recommendations: The manuscript would benefit from a clearer articulation of the practical implications of the findings, and specific policy recommendations tailored to Sri Lanka's context.

***Our response – As proposed, we have discussed the implications of our findings on human intestinal nematode infections in Sri Lanka considering the preventive and control strategies. (Lines 457-531)***
