# Supplementary material for: Human intestinal nematode infections in Sri Lanka: A scoping review
Source: PLoS Negl Trop Dis. 2024 Dec 2;18(12):e0012689. doi: 10.1371/journal.pntd.0012689 (PMC11637441; doi:10.1371/journal.pntd.0012689)
Supplement: Attachment — Submitted filename: NJ_Response to reviewers.docx [file pntd.0012689.s004.docx]

***Manuscript title:* Human intestinal nematode infections in Sri Lanka: a scoping review**

***Manuscript reference:* PNTD-D-24-00539**

**Responses to reviewers’comments**

***We sincerely appreciate all three reviewers for their constructive comments and useful insights, which were found to be invaluable in improving our scoping review.***

***Response to reviewer #* 1**

Reviewer #1: Thank you for the opportunity to review your work entitled, “ Human intestinal nematode infections in Sri Lanka: a scoping review,” which explores the distribution, trends, diagnostics, control and challenges of HINI in Sri Lanka.

While I commend the efforts of the authors in bringing this study to bear, I have a few comments and concerns for consideration to potentially improve the quality and clarity of your work. Please find below.

1. Abstract -Your introduction section sets a good stage for reader engagement starting with the setting of Sri Lanka, the burden of disease of HINI, and the gaps in literature informing the study objectives. Well done. More could be mentioned in the methodology section here on how the data were synthesized from the data sources and perhaps the keywords/query search strategy to warrant a systematic scoping methodology.

***Response –We have now detailed the approach to data synthesis, in the methodology section of the abstract, outlining the processes for both the initial and comprehensive screening phases. (Lines 36-38)***

2. Also, how many articles were identified for inclusion before diving into the findings.

***Response – The number of articles included in the review, as well as those selected for assessing the pooled prevalence were specified within the abstract. (Lines 37-38)***

3.“HINIs in Sri Lanka were first described in 1888 in the annual survey report of the Principal Civil Medical Officer.” This is already established as a burden in the introduction. Its presence here adds no value to the objectives -i.e. distribution, trends, diagnostics, control and challenges of HINI in Sri Lanka.

***Response – The statement, “HINIs in Sri Lanka were first described in 1888 in the annual survey report of the Principal Civil Medical Officer.” was removed from the results section of the abstract.***

4. I commend you describe your findings too to reflect the objectives of your inquiry. The same goes for your conclusions. Overall your abstract section should tell the key details of a PRISMA-SCr-guided report done in a way that engages readers to want to dive more into your study.

***Response – We have revised the results and conclusion sections of the abstract improving alignment with the objectives and are presented in line with the PRISMA-Scr guidelines. (Lines 40-62)***

5. Introduction - Enjoyed reading this section. Pay attention to repetitions and redundancies. Also, confirm your claims are cited per author guidelines.

***Response – We have removed redundant sentences and added citations according to the journal guidelines. (Lines 84-128)***

6. Methods - “... adhering to the Preferred Reporting Items for Systematic Reviews and Meta-Analyses extension for Scoping Reviews (PRISMA-Scr).” I don’t think this statement is accurate. The frameworks were built upon one another as described but the PRISMA-Scr was designed by Tricco and colleagues as a reporting guideline and not a framework that Arksey and co needed to adhere to. The 5 steps you identified are Arksey and O’Malley’s reporting formats and not the PRISMA-Scr reporting format. I will advise you to pick one and stick to it rather than conflate both as you did.

***Response – We have revised the methodology clarifying the use of reporting system. (Lines161-168)***

7. Did I miss the section on ensuring rigor, and quality assurance in your article selection? What about critical appraisals and evaluations of included articles? What has that done? If not why?

***Response - The quality of all prevalence assessment studies from the past two decades was evaluated using the modified Newcastle-Ottawa Quality Assessment tool, ensuring the reliability of the data presented in the study (Lines 242-249 and Supplementary file, Table 2)***

***Response to reviewer #* 2**

Reviewer #2: This is an interesting paper in an important area.

1. General comments - In this paper, you only provided data on the prevalence of any intensity of infections, which can be used to stop preventive chemotherapy when this prevalence is less than 2%. However, to declare the elimination of HINIs as a public health problem, defined as the prevalence of moderate-to-heavy intensity of infections less than 2%. There is no reported data on that in your manuscript. Can you provide this? In cases where there is no data, you mentioned it in the text.

***Response – Several articles did not provide infection intensity data. Among those that did, the classifications varied: some reported the mean eggs per gram level for each species, while others categorized intensity as light, moderate, and heavy, or light, moderate to heavy, and heavy. Due to these inconsistent classifications and the lack of comprehensive data, a precise assessment of prevalence of infection intensities could not be performed. However, we have now added a paragraph with this information at the end of the ‘Prevalence and communities affected’ sub-section. (Lines 375-381)***

2. Line 33: You mentioned the end period of the search, which was September 2022. Can you provide the starting period?

***Response – It is from the inception, and we have now included this information. (Line 33)***

3. Line 495: You mentioned less attention was given to studying intestinal nematodes like E. vermicularis and S. stercoralis without further recommendation. Could you add a recommendation to include S. stercoralis in preventive as recommended by WHO in its new roadmap over 2021 – 2030 for the control of soil-transmitted helminths?

***Response- We have included recommendations concerning the status of strongyloidiasis in Sri Lanka, considering the WHO 2021-2030 road map to combat neglected tropical diseases. (Lines 522-528)***

4. Line 496: You stated that prevalence assessment has not incorporated molecular methods that offer greater sensitivity. Could you provide more details on that aspect? Is that a feasibility issue in terms of cost-efficiency compared to the traditional diagnostic method?

***Response – We have now outlined the potential reasons for not conducting molecular prevalence assessment studies to evaluate the prevalnce of HINIs in Sri Lanka. (Lines 528-533)***

***Response to reviewer 3***

Reviewer #3: A pertinent review which highlights trends of common intestinal helminth infections in a background of improving living conditions.

1. Line 80 - infected to be replaced with 'infective'

***Response – Revised as suggested. (Line 92)***
